# Effect of kinesio taping in combination with vibration treatment on college students’ delayed-onset muscle soreness

**DOI:** 10.3389/fphys.2023.1058637

**Published:** 2023-01-25

**Authors:** Ning Que

**Affiliations:** Faculty of Table Tennis, Badminton and Tennis, Chengdu Sport University, Chengdu, China

**Keywords:** delayed-onset muscle soreness, kinesio taping, vibration treatment, pain, muscular strength

## Abstract

**Objective:** Kinesio taping (KT) and vibration treatment (VT) can alleviate delayed-onset muscle soreness (DOMS) to some extent. However, the literature reports on the difference between the two treatments, and whether a joint intervention (JI) works better than single treatments remains unknown. This study compares the effects of KT, VT and JI on DOMS in college students.

**Methods:** A total of 88 college students were randomly divided into the KT (KTG, n = 21), VT (VTG, n = 22), JI (JIG, n = 23) and control (CG, n = 22) groups. All subjects underwent DOMS moulding. The baseline; immediate and 24, 48 and 72 h visual analogue scale (VAS) scores and knee extensor maximum isometric voluntary contraction (MIVC) were determined.

**Results:** The intergroup comparison showed the following results. 1) The VAS scores of the four groups peaked at 24 h and gradually decreased. The immediate, 24 h and 48 h VAS scores followed the order: JIG > KTG and VTG > CG. The 72 h VAS score followed the order: KTG < VTG < JIG < CG. 2) The knee extension MIVC in the four groups was lowest at 24 h and then gradually increased. JIG had larger immediate MIVC than CG. KTG, VTG and JIG had larger 24 h MIVC than CG. JIG had larger 48 h MIVC than KTG and CG.

**Conclusion:** KT and VT can reduce muscle pain and strength loss caused by DOMS to varying degrees. VT is better than KT in improving pain. The combined intervention worked better than single interventions.

## 1 Introduction

Delayed-onset muscle soreness (DOMS) occurs from 8 h to 24 h after unaccustomed or intense centrifugal exercise and can peak and gradually relieve within 48–72 h, requiring approximately 1 week to restore (Afonso et al., 2021; Fleckenstein et al., 2021; Sonkodi et al., 2021; Torre et al., 2021). DOMS comes with a series of muscle structural, histological and biochemical changes, thus affecting physical fitness and exercise training effects. The increased risk of exercise injury (Akinci et al., 2020; Pupo et al., 2021) and reduced adverse effects caused by DOMS deserve concern.

In 1973, Dr. Kenso Kase invented the kinesio taping (KT) technology (Tran et al., 2021; Turgut et al., 2021). Previous studies confirmed the positive effect of KT on DOMS. KT intervention before DOMS moulding reduces bicep soreness in young men and accelerates the maximum isometric voluntary contraction (MIVC) recovery in muscles (Lee et al., 2015). KT intervention lowers the cold and thermal pain thresholds and the visual analogue scale (VAS) score for young male biceps brachii (DOMS) for 24 h (Bae et al., 2014). KT intervention also reduces centrifugal pain in the rectus femoris and hamstring (DOMS) for 24 and 168 h and improves the performance of hamstrings (Haksever et al., 2016). Furthermore, a meta-analysis suggested that appropriate vibration treatment (VT) can relieve DOMS symptoms (Lu et al., 2019). VT significantly reduces the VAS score and MIVC in ordinary young women (elbow DOMS, 50 Hz, 5 min) (Imtiyaz et al., 2014). The joint activity of ordinary male college students is increased (elbow DOMS, 20 Hz, 30 min), and the immediate VAS score (Lau and Nosaka, 2011) is reduced. MIVC for ordinary young males/females is increased (radial wrist extensor DOMS, 20 Hz, 2 min) (Koh et al., 2013). The VAS score of ordinary male college students is reduced (DOMS, 20 Hz–45 Hz, 10 min) (Wheeler and Jacobson, 2013). The above two methods can alleviate DOMS to some extent. KT can lift the skin of the ligation site, increase the tissue space and accelerate the blood and lymphatic circulation and inflammatory response (Tran et al., 2021). VT increases muscle discharge, local muscle temperature and blood flow to the skin; accelerates blood and lymphatic circulation and alleviates inflammatory response and pain (Lau and Nosaka, 2011; Koh et al., 2013; Wheeler and Jacobson, 2013; Imtiyaz et al., 2014; Lu et al., 2019). However, the literature only reports on the difference between the two treatments, and whether a joint intervention works better than single treatments remains unknown. Compared with single treatment (KT or VT), joint intervention may be a better way to delay DOMS.

This study explores the effect of KT with VT on DOMS symptoms in college students and provides the basis for the combined KT and VT intervention to reduce exercise-induced DOMS. The study hypothesises that the combination of KT and VT is more effective in relieving DOMS symptoms than a single treatments.

## 2 Materials and methods

### 2.1 Participants

This study was approved by our school’s ethics committee (2021.011). Special male college badminton students were recruited during the holidays. The inclusion criteria were as follows: students (1) between 18 and 20 years old, (2) weighing 60 kg–70 kg, (3) exercising irregularly for the last 2 weeks and (4) who complied with the Declaration of Helsinki and provided informed consent. The exclusion criteria were as follows: students with (1) motor injury to the lower limbs, (2) heart pacemaker installation, (3) cardiovascular disease and (4) epilepsy or other central nervous system diseases.

**FIGURE 1:**
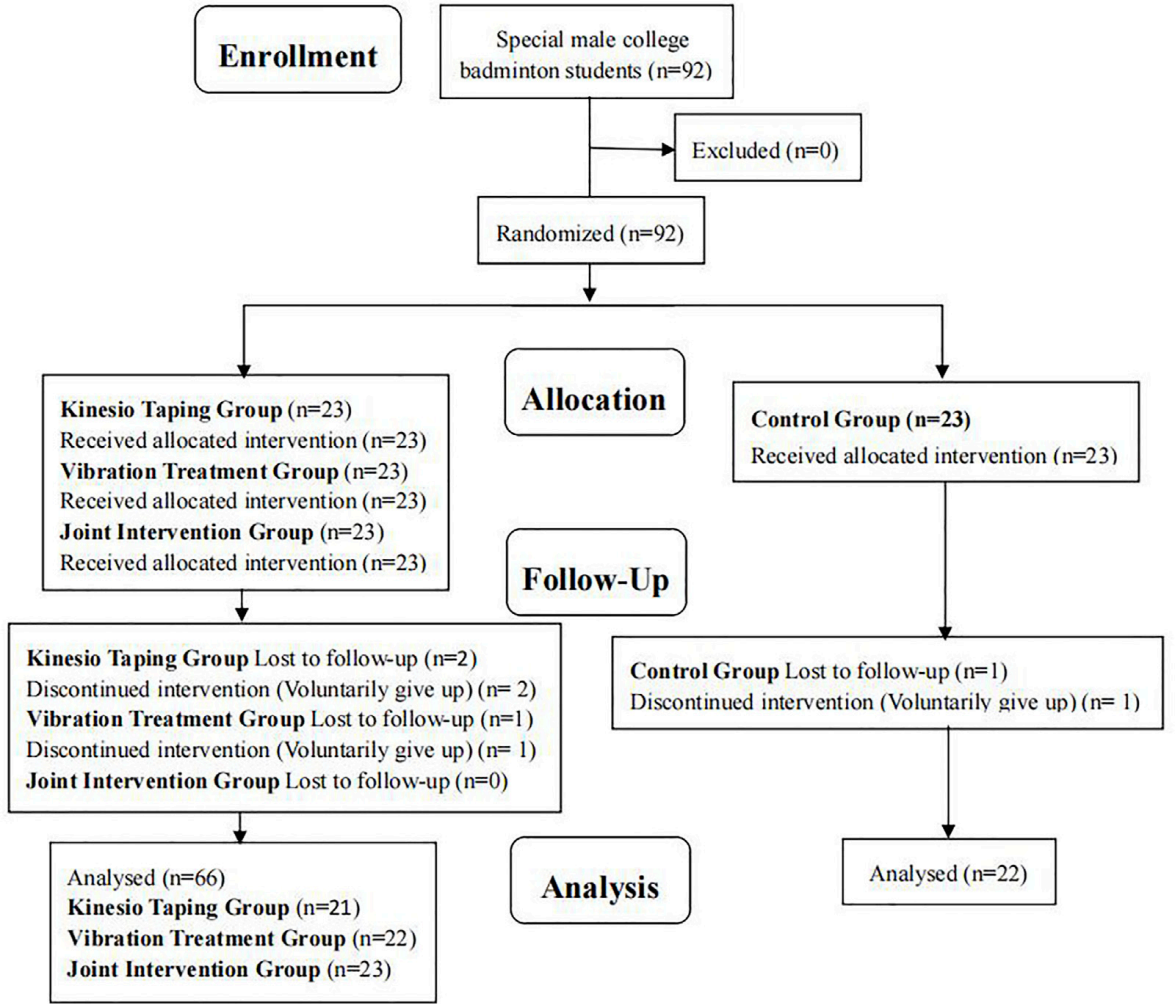
Participant selection flow diagram.

This study based the sample size on previous research results regarding the previous study on Kinesio Taping and Vibration Treatment in College Students’Delayed-Onset Muscle Soreness (Imtiyaz et al., 2014; Haksever et al., 2016), a 4 (groups) × 5 (measurement times) experimental design and a 10% sample wastage rate. G-power program was applied to calculate the effect size and total sample size. With an effect size of 0.3, α level of 0. 05 and power of 0.8, a sample size of at least 84 samples was needed. Ninety-two subjects were recruited. Four cases did not continue because of personal reasons. A total of 88 subjects completed the whole experiment (Figure 1). The random-number distribution method was used to divide subjects into four groups, i.e., KT (KTG), VT (VTG), joint intervention (JIG) and control (CG) groups. No significant difference in age, height and body weight was observed amongst groups (*p* > 0.05, Table 1). For VTG, VT was given after DOMS moulding. For KTG, Y-shaped binding was conducted for 15 min before DOMS moulding (Kirmizigil et al., 2019). After moulding, when VTG performed vibration intervention, KTG sat and rested. JIG, KTG and VTG interventions were simultaneous. For CG, no intervention happened except DOMS moulding.

**TABLE 1:** Participants’ basic information.

Group	Age (years)	Height (cm)	Weight (kg)	BMI (kg/m^2^)	VAS score	MIVC (Nm)
KTG (*n* = 21)	19.4 ± 0.2	176.1 ± 4.7	66.5 ± 4.1	21.4 ± 4.4	0.13 ± 0.03	191.2 ± 25.7
VTG (*n* = 22)	19.5 ± 0.2	176.0 ± 5.3	66.7 ± 3.8	21.5 ± 7.2	0.12 ± 0.08	193.4 ± 32.6
JIG (*n* = 23)	19.6 ± 0.2	176.3 ± 5.0	67.1 ± 3.9	21.6 ± 4.5	0.14 ± 0.08	188.2 ± 41.4
CG (*n* = 22)	19.5 ± 0.2	175.9 ± 4.6	66.3 ± 3.6	21.4 ± 4.1	0.15 ± 0.06	190.1 ± 23.5
F	0.332	0.215	0.423	0.341	0.292	0.354
P	0.769	0.823	0.661	0.711	0.746	0.692

Notes: KTG: kinesio taping group; VTG: vibration treatment group; JIG: joint intervention group; CG: Control group. VAS: Visual analog scale. MIVC: Maximum Isometric Voluntary Contraction.

### 2.2 DOMS moulding

**FIGURE 2:**
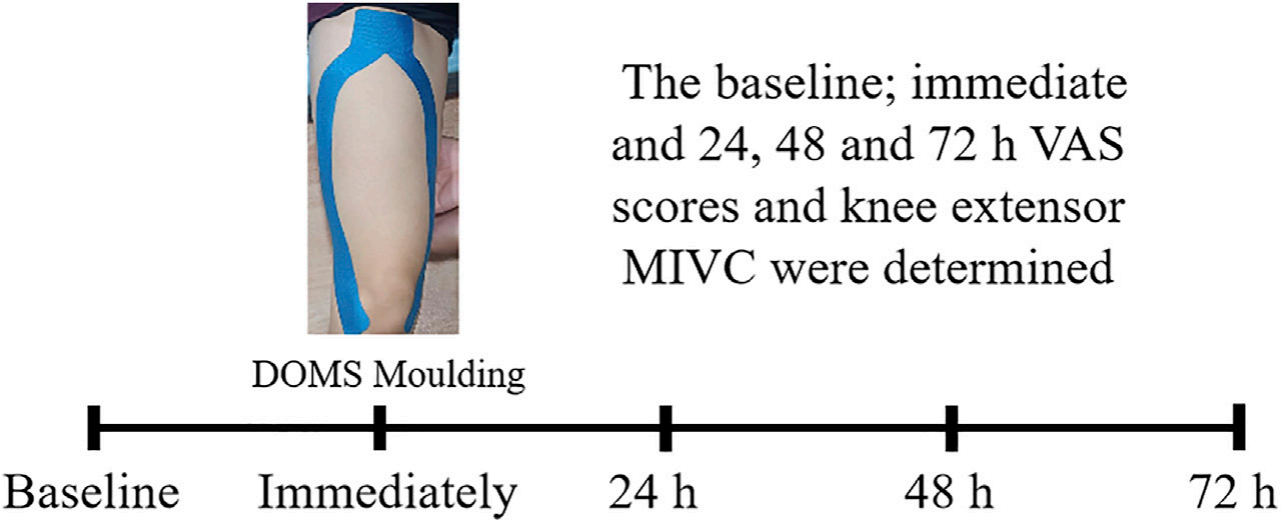
Intervention process.

In accordance with the previous method of DOMS knee moulding in the sports population (Pumpa et al., 2011; Song and Yang, 2022), all subjects underwent a maximum oxygen intake test 1 week before the moulding process (German Cortex cardiopulmonary function tester model: Metalyzer 3B). Moulding happened after a week, and subjects ran downhill on the treadmill (American ICON, model: 59916/350i; −10° grade, five groups of 8 min, with 2 min flat walking between groups). Each subject’s exercise intensity was matched on the basis of the maximum oxygen intake, ensuring that all subjects maintained similar intensity while completing the downhill run. All participants were monitored to maintain their intensity at 80% of their maximum heart rate (Model No. M430; Polar, Finland) during downhill running. The treadmill speed was appropriately adjusted if the heart rate was excessively low or high. All participants were followed up within 72 h of DOMS moulding to ensure no other training or treatment (Figure 2).

### 2.3 Kinesio taping

The “Y” ligation of the bilateral knees of KTG and JIG was conducted by a professional physical therapist 15 min before DOMS moulding (Kirmizigil et al., 2019) (KT was provided by the LP Support company, specification: 5 cm × 5 m, color: blue), stretched to 125% of the original length (Boobphachart et al., 2017). (Figure 2). To prevent KT shedding throughout DOMS moulding and within 72 h, we checked immediately and at 24, 48 and 72 h with the same ligation.

### 2.4 Vibration treatment

Under the guidance of the experimenter, the American Power-Plate^®^ pro5™ (71-PR5-3100, Frequencies 25–50 Hz) was used for 10 min on VTG and JIG (squatting and launching on the vibrational platform), referring to the vibration frequency interval and amplitude where VT had a positive effect on DOMS (Lau and Nosaka, 2011; Koh et al., 2013; Wheeler and Jacobson, 2013; Imtiyaz et al., 2014; Lu et al., 2019). This study’s vibration frequency and amplitude were 45 Hz and 3 mm (G-Factor 7 45 Hz/High Amplitude), respectively. Four groups of subjects underwent VAS score and knee extension maximum isometric muscle strength tests at DOMS baseline or before moulding and after moulding, 24, 48 and 72 h.

### 2.5 VAS score

The VAS score was used to evaluate the pain of the DOMS (quadriceps femoris) to allow the subject to draw a vertical line on a 10 cm line (0 cm, painless; 10 cm, most painful; 0–10 and left and right knee averaging) (Cheng and Jiang, 2020; Arieh et al., 2022).

### 2.6 MIVC test for knee extension

The MIVC test was performed on left and right knee joints. The subject took a seated position, and the trunk and hip joints were fixed using a wide band. During the test, the subject underwent MIVC at knee flexion 30°, and the subject makes full effort (using the maximum force as far as possible) to extend the knee joint. The test time is 15 s, at this time, the maximum peak torque recorded by the isokinetic instrument is MIVC. The left and right knees of each subject were tested. The analysis index was peak torque (Cheng and Jiang, 2020; Cheng et al., 2021), and the left and right knee data of all subjects were averaged.

### 2.7 Statistical analysis

SPSS 19.0 software, mean ± standard deviation for four groups. A two-way analysis of variance with mixed design was used to test whether groups, the main effect of time, and group (4) and time (5) interacted. If group and time interacted, the one-way ANOVA with repeated measures would vary at different time points. If time had the main effect, different time points were compared. If groups had a main effect, differences between groups were compared. The Bonferroni for posthoc comparisons was adjusted to ensure that the overall type I rate per ANOVA was not greater than 0.05. The significance level was 0.05.

## 3 Results

The results of five time points for the four subjects are shown in Table 2; Table 3. First, the two-way ANOVA VAS score and knee extension MIVC group × time interacted (*p* < 0.01), showing differences at different time points within the one-way ANOVA group. The comparison of baseline data between groups showed that VAS scores (F_(3, 84)_ = 0.292, *p* = 0.746) and MIVC (F_(3, 84)_ = 0.354, *p* = 0.692) were not significantly different (*p* > 0.05, Table 1).

**TABLE 2:** VAS scores test for knee extension at different times.

Group	Baseline	Immediately	24 h	48 h	72 h
KTG (*n* = 21)	0.13 ± 0.03	1.86 ± 0.63^ac^	2.30 ± 1.01^ac^	1.28 ± 0.43^aC^	0.60 ± 0.13^BC^
VTG (*n* = 22)	0.12 ± 0.08	1.81 ± 0.70^ac^	2.25 ± 0.89^ac^	1.15 ± 0.31^aC^	0.35 ± 0.11^Ac^
JIG (*n* = 23)	0.14 ± 0.08	1.60 ± 0.56^A^	1.72 ± 0.70^A^	0.72 ± 0.29^A^	0.13 ± 0.09^A^
CG (*n* = 22)	0.15 ± 0.06	2.30 ± 0.61	2.62 ± 1.20	1.76 ± 1.00	0.72 ± 0.26
F	0.867	6.117	5.785	11.233	26.559
P	0.928	<0.01	0.003	<0.01	<0.01

Notes: VAS: visual analog scale; KTG: kinesio taping group; VTG: vibration treatment group; JIG: joint intervention group; CG: control group. KTG, VTG, and JIG, were compared at the same time points as CG: ^a^ *p* < 0.05; ^A^ *p* < 0.01; KTG, was compared at the same time points as VTG; ^B^ *p* < 0.01. KTG, and VTG, were compared at the same time points as JIG: ^c^ *p* < 0.05;.^C^ *p* < 0.01.

**TABLE 3:** MIVC Test for Knee Extension at different times. (Nm).

Group	Baseline	Immediately	24 h	48 h	72 h
KTG (*n* = 21)	191.2 ± 25.7	177.7 ± 28.9	169.2 ± 27.8^a^	176.2 ± 26.4^c^	187.6 ± 36.2
VTG (*n* = 22)	193.4 ± 32.6	173.6 ± 25.4	172.6 ± 32.5^a^	178.1 ± 34.0	190.5 ± 28.7
JIG (*n* = 23)	188.2 ± 41.4	184.2 ± 23.0^a^	180.9 ± 28.7^a^	190.0 ± 27.5^a^	190.2 ± 38.6
CG (*n* = 22)	190.1 ± 23.5	168.2 ± 27.1	160.5 ± 21.2	177.3 ± 26.8	186.3 ± 21.0
F	0.725	2.868	2.919	2.665	0.362
P	0.830	0.020	0.013	0.032	0.701

Notes: MIVC: maximum isometric voluntary contraction; KTG: kinesio taping group; VTG: vibration treatment group; JIG: joint intervention group; CG: Control group. VAS: visual analog scale. KTG, VTG, and JIG, were compared at the same time points as CG: ^a^ *p* < 0.05; KTG, and VTG, were compared at the same time points as JIG: ^c^ *p* < 0.05.

**VAS score:** Table 2 shows that the VAS score of the four groups peaked and gradually decreased at 24 h after DOMS moulding. The comparison between groups showed the following results. KTG and VTG had lower immediate, 24 h and 48 h VAS scores than CG (*p* < 0.05). JIG had lower immediate, 24 h and 48 h VAS scores than CG (*p* < 0.01). KTG and VTG had higher immediate, 24 h and 48 h VAS scores than JIG (*p* < 0.05). VTG and JIG had lower 72 h VAS score than CG (*p* < 0.01). KTG had higher 72 h VAS score than VTG (*p* < 0.01). KTG (*p* < 0.01) and VTG (*p* < 0.05) had higher 72 h VAS score than JIG.

**MIVC:** Table 3 shows that the MIVC of the four groups was lowest at 24 h after DOMS moulding. The comparison between groups showed the following results. JIG had larger immediate MIVC than CG (*p* < 0.05). KTG, VTG and JIG had larger 24 h MIVC than CG (*p* < 0.05). JIG had larger 48 h MIVC than KTG and CG (*p* < 0.05).

## 4 Discussion

This study seeks to explore pain and MIVC changes caused by KT and VT on special college badminton students’ DOMS and provides the basis for reducing exercise-induced DOMS. We have tested the hypothesis that the combination of KT and VT is more effective in alleviating DOMS symptoms than single treatment.

### 4.1 Kinesio taping

This study found that KT significantly reduces immediate, 24 h and 48 h VAS scores. This finding confirms the results of previous similar studies on KT, which show that biceps brachii and DOMS moulding reduce immediate, 24 h, 48 h (Bae et al., 2014) and 72 h (Lee et al., 2015) VAS scores. KT is equally effective when subjects are female, and the DOMS site is the thigh, thus reducing the VAS score for the rectus femoris and hamstrings at 48 and 168 h in young women (Haksever et al., 2016).

Why does KT reduce muscle pain caused by DOMS? The vessel diameter of the DOMS site decreases, thus impairing blood flow reaction and causing hyperalgesia (Ko and Clarkson, 2020). Tissue swelling at the DOMS damage site affects blood and lymph circulation (Fang and Nasir, 2021). KT can lift the skin of the ligation site, increase the tissue space and accelerate the blood and lymphatic circulation and the inflammatory response. This lifting can help relieve pressure on nociceptors directly under the skin and remove the accumulated metabolites (Tran et al., 2021), thus reducing pain and feeling. Alternatively, KT provides sustained tactile and proprioceptive inputs to the ligation site, thereby inhibiting the input of nociception (Saki et al., 2022).

This study found that KT significantly enhances the MIVC of the knee extensor for 24 h. The meta-analysis (including four randomised controlled trials) confirmed our conclusion that KT significantly increases the knee extensor muscle (DOMS) MIVC (Tran et al., 2021). KT increases skin input then enhances the function of neuromuscles, promotes muscle activity at the ligation site, improves muscle structure and contributes to a slight increase in muscle force (Tran et al., 2021). Furthermore, the subject reduces the muscle strength test caused by pain (does not exert more force).

### 4.2 Vibration treatment

This study found that VT significantly reduces immediate, 24 h, 48 h and 72 h VAS scores. Previous studies showed that VT reduces muscle pain in the knee joint immediately after DOMS. VT significantly reduces the immediate, 24 h and 48 h VAS scores (20 Hz–45 Hz, 10 min) of ordinary college male students (Wheeler and Jacobson, 2013) and significantly reduces the VAS scores (30 Hz, 10 min) of men at 96 and 120 h (Broadbent et al., 2010). In the present study, VT at 45 Hz for 10 min is adopted for DOMS sites. The vibration frequency and time of the intervention vary, and the VAS score is significantly reduced at different measurement times. These findings are similar to those of previous studies (Broadbent et al., 2010; Wheeler and Jacobson, 2013), showing that VT can relieve muscle pain after DOMS moulding.

Several current views explained the pain reduction caused by VT. VT stimulates myospindle and motor neurons, causing strong muscle contraction to produce a perceptual response and relieve pain. VT activates large-muscle diameter fibres, inhibits small-diameter fibres and reduces pain. VT also accelerates the stimulation of inhibitory interneurons in the middle nerve endings of the spinal nerve and reduces the transmission of perceptual information from the spinal cord to the brain (feedback of proprioceptors for nociception inhibition) to reduce pain. VT increases muscle discharge, local muscle temperature and blood flow to the skin; accelerates blood and lymphatic circulation and alleviates the inflammatory response and pain (Lau and Nosaka, 2011; Koh et al., 2013; Wheeler and Jacobson, 2013; Imtiyaz et al., 2014; Lu et al., 2019).

VTG has significantly higher MIVC than CG (24 h). In previous studies, VT (50 Hz, 5 min) on the elbow in ordinary young women shows that the MIVC increases at 72 h (Koh et al., 2013). VT (50 Hz, 30 min) on common male/female femoral quadriceps reveals a significant increase in MIVC for 24 h (Bakhtiary et al., 2007). The above study partly supports the present study’s conclusions. The analysis may be related to the activation of increased motor units (DOMS sites) in increased muscle tension. Muscle spindle stimulation by vibration may increase the afferent activities of muscle spindles, which may increase background tension in the vibrated muscles (Bakhtiary et al., 2007; Broadbent et al., 2010; Koh et al., 2013). Furthermore, given the reduced muscle pain, the subject is believed to have reduced muscle strength caused by pain (afraid to force) during the isometric muscle force test.

### 4.3 Joint intervention

This study has compared KTG, VTG and JIG. The VAS score of JIG at 72 h decreases significantly compared with that of the control group. The MIVC of the extensor knee muscles is significantly increased at 24 and 48 h. The VAS scores of JIG immediate ∼72 h are considerably smaller than those of KTG and VTG, indicating that the combined intervention can better relieve the muscle pain caused by DOMS than single treatments. The MIVC of JIG is significantly higher than that of KTG (48 h), indicating that the combined intervention significantly affects muscle force at 48 h than single treatments. No literature report has been made on combining conditions on muscle pain and strength at the DOMS site. The effects are also speculated to increase after the joint intervention of KT and VT, and we can provide evidence for similar future studies. This study has found that the VAS score (72 h) of VTG is less than that of KTG, indicating that VT relieves muscle pain caused by DOMS better than KT over time. Our results suggest that, in clinical practice, reasonable KT and VT intervention may delay the symptoms of DOMS when the exercise population performs high-intensity exercise.

This study has some limitations. First is the small sample size, which only comprise special college badminton students. Second, no blood indicator is collected. Lastly, a placebo treatment is not designed. Further expansion of the sample is needed to explore how KT and VT improve DOMS symptoms.

## 5 Conclusion

KT and VT can significantly reduce muscle pain and strength loss caused by DOMS. VT works better than KT in improving pain. The combination treatment is better than single interventions.

## Data Availability

The raw data supporting the conclusion of this article will be made available by the authors, without undue reservation.
